# Diagnostic yield of simultaneous dynamic contrast-enhanced magnetic resonance perfusion measurements and [^18^F]FET PET in patients with suspected recurrent anaplastic astrocytoma and glioblastoma

**DOI:** 10.1007/s00259-022-05917-3

**Published:** 2022-07-30

**Authors:** Otto M. Henriksen, Adam E. Hansen, Aida Muhic, Lisbeth Marner, Karine Madsen, Søren Møller, Benedikte Hasselbalch, Michael J. Lundemann, David Scheie, Jane Skjøth-Rasmussen, Hans S. Poulsen, Vibeke A. Larsen, Henrik B. W. Larsson, Ian Law

**Affiliations:** 1 Dept. of Clinical Physiology Nuclear Medicine and PET, Copenhagen University Hospital Rigshospitalet, Copenhagen, Denmark; 2 Dept. of Radiology, Copenhagen University Hospital Rigshospitalet, Copenhagen, Denmark; 3 Dept. of Clinical Medicine, Faculty of Health and Medical Science, University of Copenhagen, Copenhagen, Denmark; 4 Dept. of Oncology, Copenhagen University Hospital Rigshospitalet, Copenhagen, Denmark; 5 Dept. of Clinical Physiology and Nuclear Medicine, Copenhagen University Hospital Bispebjerg, Copenhagen, Denmark; 6 Dept. of Pathology, Copenhagen University Hospital Rigshospitalet, Copenhagen, Denmark; 7 Dept. of Neurosurgery, Copenhagen University Hospital Rigshospitalet, Copenhagen, Denmark

**Keywords:** Glioma, Magnetic resonance imaging, Perfusion imaging, Blood volume, Positron emission tomography, Amino acid tracers

## Abstract

**Purpose:**

Both amino acid positron emission tomography (PET) and magnetic resonance imaging (MRI) blood volume (BV) measurements are used in suspected recurrent high-grade gliomas. We compared the separate and combined diagnostic yield of simultaneously acquired dynamic contrast-enhanced (DCE) perfusion MRI and O-(2-[^18^F]-fluoroethyl)-L-tyrosine ([^18^F]FET) PET in patients with anaplastic astrocytoma and glioblastoma following standard therapy.

**Methods:**

A total of 76 lesions in 60 hybrid [^18^F]FET PET/MRI scans with DCE MRI from patients with suspected recurrence of anaplastic astrocytoma and glioblastoma were included retrospectively. BV was measured from DCE MRI employing a 2-compartment exchange model (2CXM). Diagnostic performances of maximal tumour-to-background [^18^F]FET uptake (TBR_max_), maximal BV (BV_max_) and normalised BV_max_ (nBV_max_) were determined by ROC analysis using 6-month histopathological (*n* = 28) or clinical/radiographical follow-up (*n* = 48) as reference. Sensitivity and specificity at optimal cut-offs were determined separately for enhancing and non-enhancing lesions.

**Results:**

In progressive lesions, all BV and [^18^F]FET metrics were higher than in non-progressive lesions. ROC analyses showed higher overall ROC AUCs for TBR_max_ than both BV_max_ and nBV_max_ in both lesion-wise (all lesions, *p* = 0.04) and in patient-wise analysis (*p* < 0.01). Combining TBR_max_ with BV metrics did not increase ROC AUC. Lesion-wise positive fraction/sensitivity/specificity at optimal cut-offs were 55%/91%/84% for TBR_max_, 45%/77%/84% for BV_max_ and 59%/84%/72% for nBV_max_. Combining TBR_max_ and best-performing BV cut-offs yielded lesion-wise sensitivity/specificity of 75/97%. The fraction of progressive lesions was 11% in concordant negative lesions, 33% in lesions only BV positive, 64% in lesions only [^18^F]FET positive and 97% in concordant positive lesions.

**Conclusion:**

The overall diagnostic accuracy of DCE BV imaging is good, but lower than that of [^18^F]FET PET. Adding DCE BV imaging did not improve the overall diagnostic accuracy of [^18^F]FET PET, but may improve specificity and allow better lesion-wise risk stratification than [^18^F]FET PET alone.

**Supplementary Information:**

The online version contains supplementary material available at 10.1007/s00259-022-05917-3.

## Introduction

The prognosis of high-grade gliomas is poor, and treatment options are limited [1]. Accurate diagnosis of tumour recurrence remains a challenge in patients with treated high-grade glioma, and magnetic resonance imaging (MRI) thus plays a pivotal role in the post-treatment management of brain tumour patients. However, the diagnostic accuracy of conventional MRI in the post-treatment setting is low due to the presence of treatment-induced changes mimicking disease progression. Post-treatment-related effects include both pseudo-progression, an acute inflammatory response to radio-chemotherapy, and late treatment damage (radiation necrosis). Surgical trauma may further complicate the evaluation of MRI up to 2–3 months after surgery [2]. The specificity of conventional MRI for biopsy-verified recurrent glioma has been reported to be as low as 50% [3]. Various additional functional imaging modalities may be applied in order to establish the nature of progressive MRI lesions, e.g. MRI perfusion measurements and positron emission tomography (PET) [4].

MRI perfusion measurements for estimation of tumour blood volume (BV), considered a measure of tumour angiogenesis, are most commonly performed using the dynamic susceptibility contrast (DSC) approach [5]. Although high diagnostic accuracy with sensitivity and specificity in the 85–90% range for detection of progressive disease has been reported [6], the DSC approach is limited by being non-quantitative and suffers from incomplete coverage of lesions in the presence of susceptibility artefacts often present in the post-treatment setting [7–9]. The alternative T1 weighted dynamic contrast-enhanced (DCE) approach allows conversion of the MRI signal to gadolinium (Gd) concentrations for kinetic modelling and is, in addition, less affected by susceptibility artefacts [9, 10]. A meta-analysis of studies of recurrent glioma found high pooled sensitivity (89%) and specificity (85%) of DCE to be similar to that of DSC when using the best-performing DCE parameter of the individual studies [6]. These previous studies were based on kinetic modelling using 2 or 3 parameter models (Tofts’ or the extended Tofts’ model) [11], or on model-independent area under signal curve-derived metrics [12]. Advances in DCE MRI allow sampling with higher temporal resolution and thus quantification of blood flow (F) which in turn permits the use of 4-parameter 2-compartment exchange models (2CXM), providing quantification of permeability (Ki) and blood volume (BV) [13–15]. To our knowledge, the diagnostic accuracy of 2CXM DCE in recurrent gliomas has not been investigated.

Amino acid PET tracers such as O-(2-[^18^F]-fluoroethyl)-L-tyrosine ([^18^F]FET) expressing L-amino-transferase on glioma cells are increasingly being used for discriminating tumour from post-treatment changes [16]. A meta-analysis of [^18^F]FET found pooled sensitivity and specificity of 90% and 85%, respectively [17], and a recent study of [^18^F]FET PET reported even higher diagnostic accuracy in late recurrent glioblastomas (> 6 months after radiotherapy) [18], Still, increased tracer uptake may be observed in post-treatment changes [19]; thus, robust methods to improve specificity and evade false-positive findings are warranted.

Several studies have compared diagnostic the performance of amino acid PET with that of DSC perfusion MRI in suspected recurrent gliomas [20–28], but comparative studies applying DCE have to our knowledge not been published.

At our institution, [^18^F]FET PET/MRI with DCE BV imaging is applied to routine clinical imaging of glioma patients and is in particular used for high-grade gliomas in the post-treatment setting for patients with possible or suspected recurrent disease due to residual or progressive MRI lesions or clinical symptoms, and in patients scheduled for second-line treatment. These unique data allow us to compare the two modalities acquired simultaneously under similar physiological conditions in a large and clinically highly relevant patient population with suspected recurrence with correlation to clinical outcome. The aims of the study were primarily to compare the diagnostic value of simplified (semi-) quantitative cut-offs from DCE perfusion MRI and [^18^F]FET PET for detection of short-term disease progression, and secondly to investigate if bi-modal advanced imaging may be more accurate than single modalities.

## Methods

### Patient population

From the image archive system, we retrospectively identified all non-paediatric hybrid [^18^F]FET PET/MRI brain scans (*n* = 542) performed between January 2016 and September 2020. Eligibility according to the below criteria was initially assessed from the indication as stated in the imaging report and subsequently confirmed from patients’ records. Retrospective use of clinical data was approved by the Danish Patient Safety Authority (reference no. 3–3013-1957/1) and for data after January 2020 by the local hospital administration (Copenhagen University Hospital Rigshospitalet). We also included baseline data from patients with RANO progression included in a study of combined nivolumab/bevacizumab undergoing surgery after a single dose of nivolumab. This prospective study was approved by the local ethics committee (The Capital Region of Denmark Committee on Health Research Ethics, ref. H-17040888) and conducted in accordance with the Helsinki Declaration, and participants gave informed written consent prior to the scan.

Inclusion criteria comprised

1. adult patients (> 18 years) with high-grade gliomas referred for [^18^F]FET PET/MRI due to findings suggestive of residual/progressive lesions on a previous MRI or progressive clinical symptoms,
2. histologically verified WHO grade III anaplastic astrocytoma (AIII) or grade IV glioblastoma (GBM) including secondary GBM according to the WHO 2016 classification [29], and
3. prior standard therapy, i.e. maximal safe resection and/or radiation/chemotherapy (please refer to Supplementary Table S1 in Online resource 1).

Exclusion criteria comprised.

1. tumours with oligodendroglial origin (1p/19q co-deletion) or atypical/mixed pathology (e.g. sarcomatous components) were not included in order to ensure a homogenous study sample,
2. previous or current exposure to antiangiogenic treatment, immune therapy or other non-standard therapy,
3. technical sub-optimal examination including a T1 measurement with less than 4 flip angles or DCE or PET imaging of poor quality (e.g. motion, poor DCE input function), and
4. non-evaluable outcome within 6 months follow-up (see below).

**Fig. 1:**
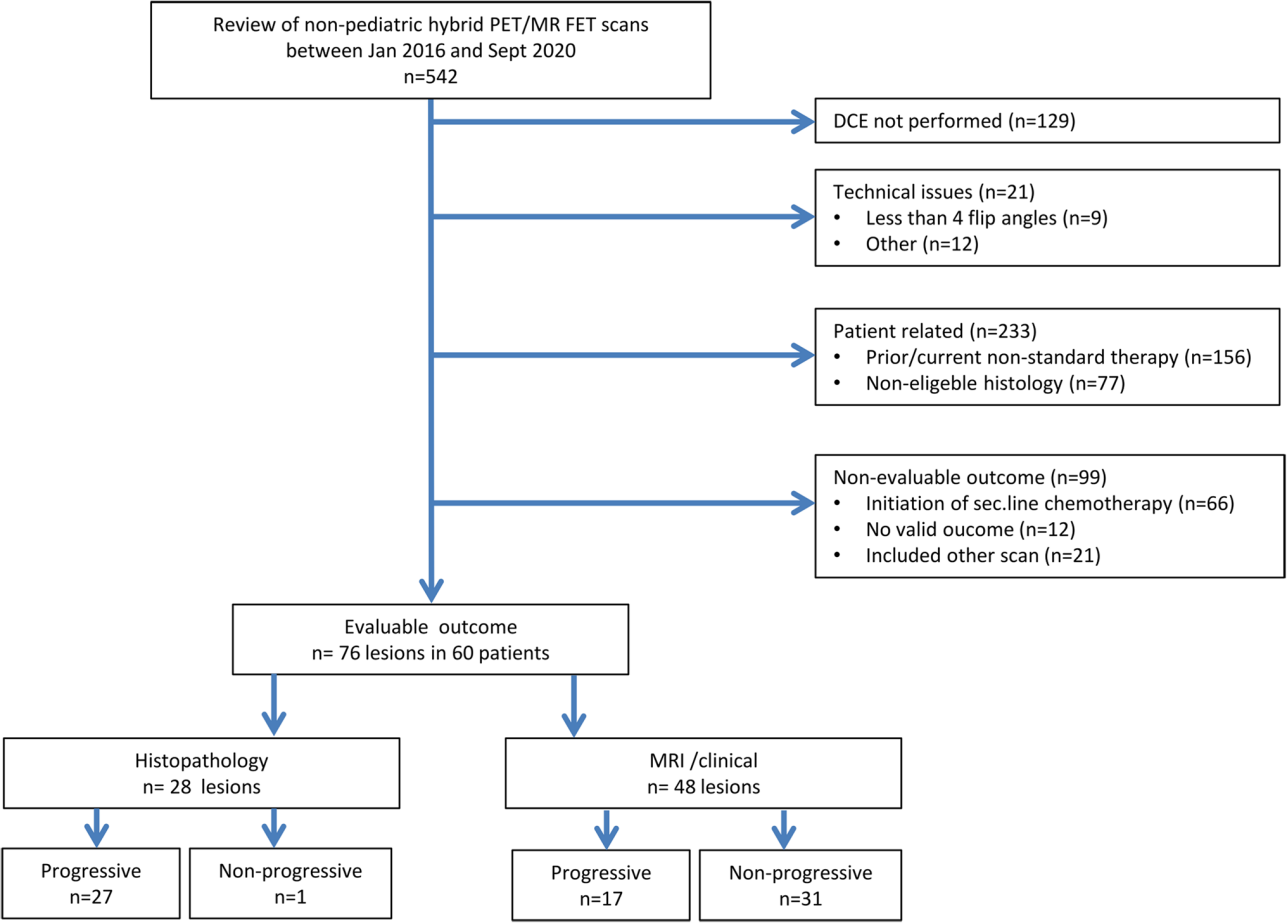
Flow chart of lesion and patient inclusion. Exclusion due to “other” technical issue includes issues related to DCE MRI (quality of input function *n* = 4, patient stopped scan *n* = 1, failure of power injector *n* = 1, calculation *n* = 2), [.^18^F]FET PET (motion *n* = 1, attenuation correction *n* = 1, delayed tracer injection *n* = 1) or both (subcutaneous injection *n* = 1). Non-eligible histology comprises oligodendroglioma (*n* = 29), ependymoma (*n* = 1), other low grade or paediatric tumours (*n* = 16), metastases (*n* = 16), pre-treatment imaging (*n* = 6), sarcoma or gliosarcoma (*n* = 8) and xantoastrocytoma (*n* = 1)

The flow chart of inclusion is provided in Fig. 1. We included a total of 76 unique lesions in 60 patients with the evaluable outcome within 6 months of follow-up as determined by histopathology in 28 lesions (27 progressive), MRI findings 45 (14 progressive) or by clinical decision in 3 (progressive) lesions. In patients with outcomes evaluated by histopathology, tissue sampling surgery/biopsy was obtained at a median of 13 days (range 7–92 days) after the scan. In one additional patient, surgery was performed 290 days after the scan due to progressive changes retrospectively visible on MRI 5 months after the PET/MRI scan. In one patient, the patient-wise outcome could not be determined. Details of lesion characteristics and lesion-wise outcome are provided in Online resource 2. In one patient with AIII, imaging was performed 4 weeks after primary surgery to assess residual tumour. All other patients had both surgery (or biopsy) and radiotherapy at some point prior to imaging.

### Pathology

Tumour classification including methylguanine-DNA methyltransferase gene (MGMT) promoter methylation and isocitrate dehydrogenase-1 (IDH) mutation status was recorded as stated in the pathology report established at the last surgery or biopsy.

### Follow-up and reference standard

Clinical records including pathology reports and subsequent imaging were reviewed at the time of data analysis (April 2021). Diagnostic reference was based on both lesion and patient-wise follow-up at 6 (± 2 weeks) months after PET/MRI imaging.

In order to allow both lesion and patient-wise analysis, the modified RANO criteria [30] were adapted to the purpose of the analysis. Progressive disease was thus defined by (1) histopathological verification on biopsy or resection, (2) progression at a follow-up MRI at least 4 weeks later (≥ 25% increase in the products of perpendicular diameters of enhancing lesions, significant progression of non-enhancing lesions, or any new enhancing lesion, or (3) clinical diagnosis of progressive disease (e.g. clear clinical deterioration not attributable to other causes apart from tumour or attributable to changes in steroid dose, or death of failure to attend follow-up attributable to progressive disease).

Non-progression was defined as the absence of tumour on histopathology, regression (> 50% reduction in the products of perpendicular diameters) or stable disease using the modified RANO criteria [30] within a follow-up period of 6 months. As both recurrent tumour and treatment-related changes respond to anti-angiogenetic second-line therapy, e.g. bevacizumab, only patients with no other chemotherapy than (continued) initial adjuvant temozolomide until the endpoint was met were included. Other reasons for exclusion were lack of confirmation in case of, e.g., death from other causes or termination of treatment without diagnostic confirmation as defined above.

In ambiguous cases, the outcome was determined by consensus among two authors (OH and IL). When criteria were conflicting, the clinical decision of progression vs non-progression as stated in the patient record was used.

In patients with multiple lesions and only clinically defined progression, only the dominant (if present) lesion was classified as progressive and included only in the lesion-wise analysis, whereas the maximal value from all lesions (also without lesion-wise outcome) was used in the patient-wise analysis. In lesions/patients with multiple scans, only data from a single PET/MR scan was included in the analysis; for lesions with histopathological confirmation, the last PET/MRI scan before biopsy/surgery was included; otherwise, the first PET/MR scan with the longest follow-up was used.

### Imaging protocol

Imaging was performed on a Siemens Biograph mMR 3 T hybrid PET/MRI system equipped with a 16-channel head coil (Siemens Biograph, Siemens, Erlangen, Germany). PET imaging was performed according to recent guidelines [31].

The hybrid PET/MRI protocol included a single-bed 20-min simultaneous PET/MRI acquisition initiated 20 min after i.v. injection of approximately 200 MBq [^18^F]FET. Details of the imaging protocol, DCE acquisition and PET reconstruction are provided in Online resource 1.

DCE data was analysed using in-house software for Matlab as previously described [13, 14, 32] in which blood flow is estimated by model-free deconvolution by Tikhonov’s method, and subsequent fitting of BV, permeability (the unidirectional clearance constant, Ki) and extra-vascular, extra-cellular space (Ve) from a 2-compartment model.

### Image analysis

Conventional pre- and post-contrast MRI were read as a part of the clinical routine by an experienced neuroradiologist unless reporting was not deemed relevant due to a very recent diagnostic MRI.

For the purpose of the study, [^18^F]FET PET and DCE BV images were re-analysed by a single author (OH) blinded to the disease course after imaging. Images were analysed using Mirada RTx software (Mirada Medical, Oxford, UK).

Images were initially analysed lesion-wise. A lesion was defined as a spatially distinct MRI T2/T2 FLAIR hyperintensity or post-contrast enhancement on T1 MRI, or as focal uptake on [^18^F]FET PET. Initially, [^18^F]FET PET and DCE parameter maps were registered and displayed fused to post-contrast T1 MRI. In order to minimise the confounding influence of [^18^F]FET PET on the reading of DCE BV maps, the images were analysed in a fixed order (see below) blinded to subsequently analysed modalities. In single cases, DCE BV was re-evaluated if [^18^F]FET PET indicated tumour components not recognised initially.

### Post-contrast-enhanced T1 MRI

Lesions were classified as enhancing in the presence of any contrast enhancement irrespective of its suspected nature or a predominant larger non-enhancing component. Guided by the MRI report, the contrast enhancement was delineated in 3D by isocontouring and adjusted manually to obtain the contrast-enhancing volume (VOL_CE_). Within VOL_CE_, the median values of blood flow (F_med_), BV (BV_med_), Ki (Ki_med_) and of median [^18^F]FET uptake to cortex ratio (TBR_med_) within VOL_CE_ were recorded in order to obtain unbiased and representative quantitative values for all parameters of interest from anatomically defined lesion volumes.

### DCE BV imaging

On BV maps co-registered to post-contrast 3D-T1 MRI, the volume of visually increased BV (VOL_BV_) was delineated by isocontouring and adjusted manually avoiding signal from visible macrovascular structures. The voxel-wise maximal BV (BV_max_) within VOL_BV_ was recorded, and the normalised maximal BV_max_ (nBV_max_) was calculated as the ratio of BV_max_ to the mean BV value of an ellipsoid VOI (approx. 1 ml) drawn in the normal-appearing white matter of the contralateral hemisphere, usually in the centrum semiovale. In visually BV negative lesion, BV_max_ was determined as the maximal value within VOL_CE_ or for non-enhancing lesions either within VOL_FET_ or, if also [^18^F]FET negative, in a spherical VOI in the centre of the FLAIR lesion.

### [^18^F]FET PET

The metabolically active [^18^F]FET volume (VOL_FET_) was delineated by isocontouring. VOL_FET_ was defined as tissue with [^18^F]FET uptake exceeding 1.6 of the mean activity of a background region drawn in the normal-appearing cortex of the contralateral hemisphere [33].

Within VOL_FET_, the maximal tumour-to-background [^18^F]FET uptake ratio (TBR_max_) was calculated as a measure of maximal metabolic activity. In [^18^F]FET negative lesions (TBR_max_ < 1.6), maximal uptake was determined in an approach similar to BV imaging.

### Statistics

For continuous parameters, median (range) is reported and group differences are tested using the Mann–Whitney test, while Wilcoxon signed rank test is used for paired observations. Categorical variables were analysed using Fischer’s exact test. A two-tailed significance level of 0.05 was applied.

Diagnostic accuracy was assessed and compared between [^18^F]FET PET and DCE using the receiver operating characteristics (ROC) area under the curve (AUC) constructed from logistic regression models using single or multiple explanatory parameters. The equality of ROC AUCs was tested using the DeLong test [34]. Empirical optimal cut-offs of TBR_max_, BV_max_ and nBV_max_ (and for median values within contrast-enhancing volumes) for separation of progressive and non-progressive lesions were derived from ROC analysis by maximisation of Youden’s index. As the exploratory analysis showed substantial differences between enhancing and non-enhancing lesions, optimal cut-off and associated sensitivity, specificity, and positive and negative predictive values were determined both for all lesions and separately for enhancing and non-enhancing lesions. To assess the diagnostic value of [^18^F]FET PET and DCE combined, the combinations of best-performing parameters and cut-offs were used. Lesions above and below the optimal cut-off for each parameter were classified as positive and negative, respectively. In combined imaging, only lesions positive on both were classified as positive, while lesions negative on either or both were classified as negative.

Subgroup analysis was performed to assess the potential influence of recent radiotherapy (< 6 months), and of IDH and MGTM status. Due to the low number of non-enhancing lesions, subgroup analyses were only performed on enhancing lesions.

All statistical analyses were performed in STATA 15 (Stata Corp, College Station, TX).

## Results

**Fig. 2:**
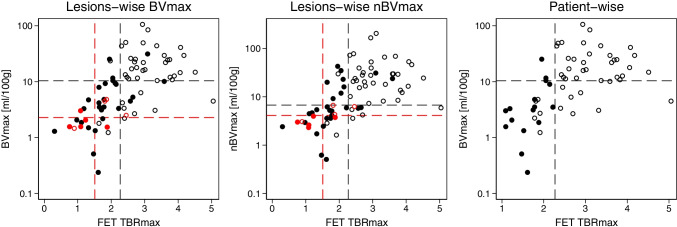
Correlation of absolute and relative blood volume with [^18^F]FET uptake according to progression. Scatterplots of absolute (left) and normalised (centre) maximal blood volume vs TBR_max_ in progressive (open, *n* = 44) and non-progressive (solid, *n* = 32) lesions. Non-enhancing lesions are shown in red. Vertical and horizontal lines show optimal cut-offs for enhancing lesions (black) and non-enhancing lesion (red). Right: scatterplot of patient-wise maximal blood volume and TBR_max_ with optimal cut-offs indicated with dashed lines in patients with progression (open, *n* = 43) and without progression (solid, *n* = 16). Note the logarithmic *y*-axis

**Fig. 3:**
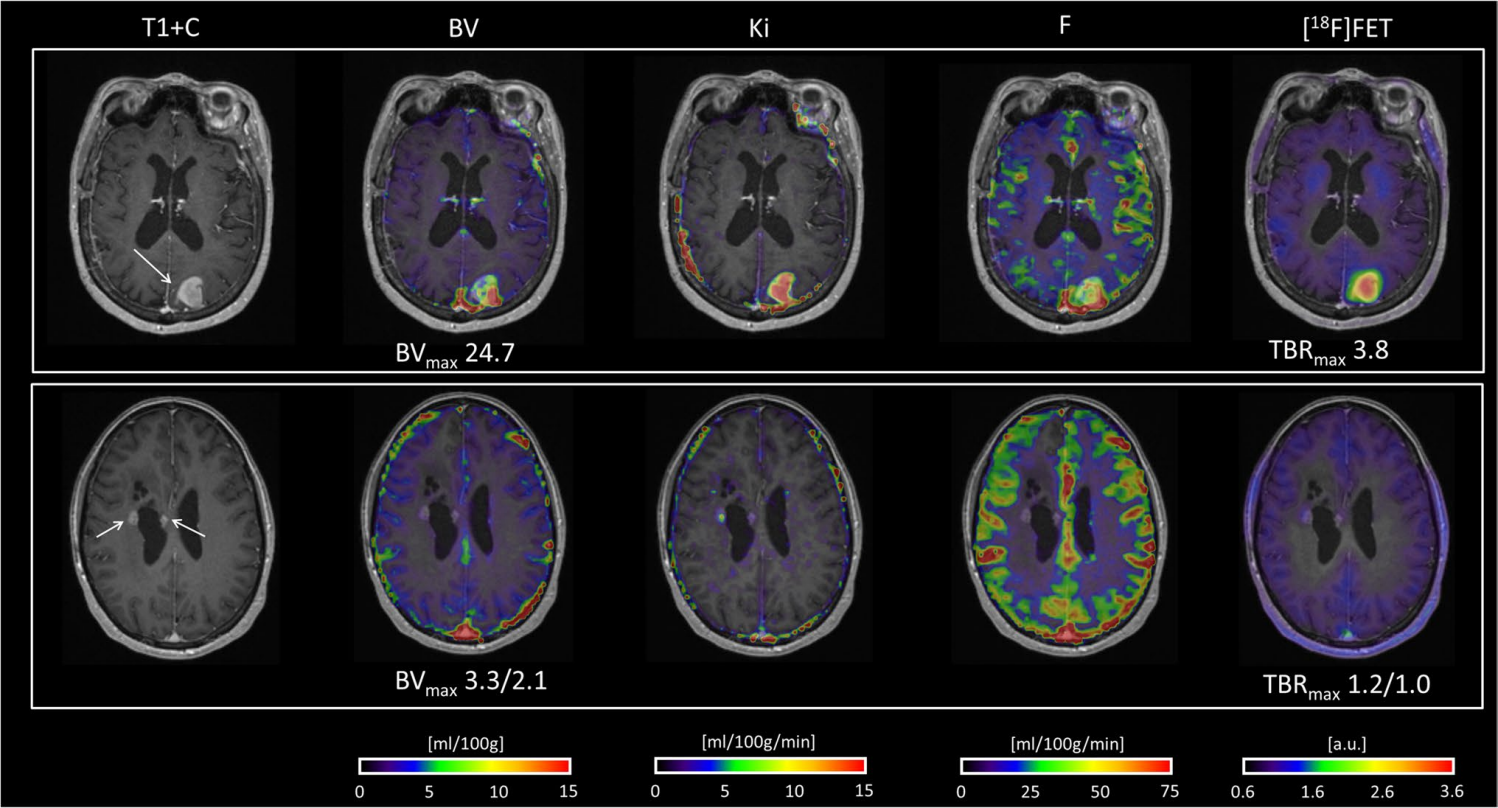
Examples of [^18^F]FET PET and DCE BV imaging providing similar information. *Upper row:* IDH-mutated GBM, progressive enhancing lesion in the left occipital lobe 7 months after surgery and radiotherapy and 1 month after completing adjuvant temozolomide. Subsequent surgery confirmed recurrent GBM. *Lower row:* IDH wild-type GBM, MRI 16 months after surgery and radiotherapy and 9 months after adjuvant temozolomide shows two stable small enhancing lesions and progressive non-enhancing signal changes (not shown) around the right lateral ventricle. PET/MRI shows increased permeability, but neither increase BV nor [^18^F]FET uptake in the two enhancing lesion. On MRI after 7 months without therapy, the lateral lesion remained stable and the medial had regressed

**Fig. 4:**
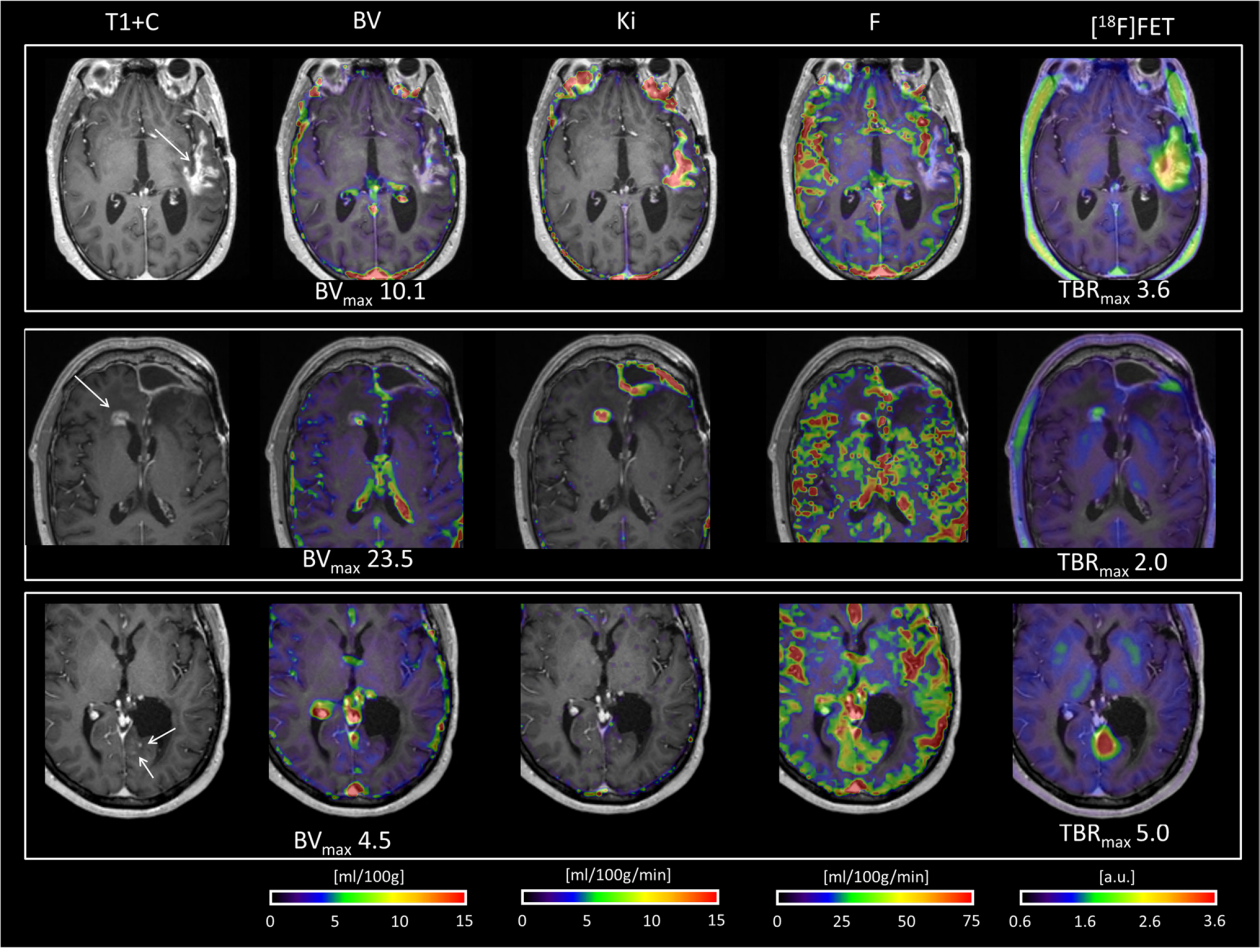
Examples of [^18^F]FET PET and DCE BV imaging providing complementary information. *Upper row*: IDH wild-type GBM, MRI 11 months after radiotherapy show a progressive large stellate enhancing lesion in the left temporal lobe. PET/MR shows high [^18^F]FET uptake and increased permeability, but only focally increased blood volume (BV) below the cut-off. The patient had a prior history of histologically confirmed pseudo-progression 5 months earlier and was followed with frequent imaging. At MRI follow-up after 2 months, the enhancing lesion had regressed. *Middle row:* IDH wild-type GBM, progressive enhancing lesion 9 months after radiotherapy in the right mesial frontal lobe. PET/MR showed mildly increased [^18^F]FET uptake below the cut-off, but clearly increased permeability and BV above the cut-off. Surgery confirmed recurrent GBM. *Lower row:* A patient with grade III astrocytoma, IDH mutated, 4 years after last surgery and radiotherapy. PET/MR was performed due to new punctate enhancing lesions (arrow) but stable non-enhancing signal changes (not shown). DCE showed mildly increased blood volume below the cut-off visually difficult to separate from the vascular signal, while [^18^F]FET uptake was markedly increased. Subsequent surgery confirmed recurrent grade III astrocytoma

Summary statistics for evaluable lesions are shown in Table 1. Overall, in 60 patients, 76 unique lesions were included, of which 67 lesions were contrast-enhancing and 9 non-enhancing. All [^18^F]FET PET, DCE and CE tumour metrics were significantly higher in progressive (*n* = 44) compared to non-progressive (*n* = 32) lesions. Scatter plots of correlations of median and maximal BV with [^18^F]FET uptake in progressive and non-progressive lesions are shown in Fig. 2. Compared to lesions with clinical/radiological follow-up, lesions with histopathological verification were, in general, larger and were both metabolically more active and had higher BV values (Supplementary Table S2 in Online resource 1). Examples of [^18^F]FET PET and DCE imaging are provided in Figs. 3 and 4.

**Table 1:** Lesion summary statistics

		All (*n* = 76)	Non-progressive (*n* = 32)	Progressive (*n* = 44)
Lesion characteristics	Days from last surgery	227 (15–2167)	282 (25–1041)	224 (15–2167)
	Days from RT	246 (56–4494)	277 (67–966)	226 (56–4494)
	GBM, *n* (%)	67 (88)	28 (88)	39 (89)
	IDH wild-type, *n* (%)	64 (84)	26 (81)	38 (86)
	MGMT methylated, *n* (%)	42 (55)	24 (75)	18 (41)‡
	RANO group	9/32/32	5/22/5	4/10/30‡
Maximum lesion values	TBR_max_	2.4 (0.3–5.0)	1.7 (0.3–3.6)	2.8 (0.9–5.0)‡
	BV_max_ (mL/100 g)	9.2 (0.2–106.3)	3.4 (0.2–31.3)	14.7 (1.2–106.3)‡
	nBV_max_	11.0 (0.5–204.8)	4.9 (0.5–42.6)	20.5 (1.6–204.8)‡
Lesion volumes	VOL_FET_ (ml)	4.2 (0–46.4)	0.06 (0–32.5)	11.2 (0–46.4)‡
	VOL_BV_ (ml)	0.4 (0–33.8)	0 (0–7.6)	2.8 (0–33.8)‡
	VOL_CE_ (ml)	1.3 (0–32)	0.3 (0–16.9)	3.4 (0–32)‡
Median values in contrast	TBR_med_	1.8 (0.3–3.1)	1.3 (0.3–2.2)	2.0 (1.2–3.1)‡
enhancing volumes	F_med_ (mL/100 g/min)	18.2 (3.1–73.4)	13.1 (3.1–54.5)	22.7 (11.2–73.4)‡
(*n* = 67)	BV_med_ (mL/100 g)	2.3 (0.14–14.7)	1.3 (0.1–7.4)	3.0 (0.4–14.7)‡
	Ki_med_ (mL/100 g/min)	6.2 (0–40)	4.3 (0–40)	6.7 (0.2–40)†

^†^*p* < 0.05 ‡*p* < 0.01 *RT*, radiotherapy; *GBM*, glioblastoma; *IDH*, isocitrate dehydrogenase; *MGMT*, methylguanine-DNA-methyltransferase; *RANO*, group (non-enhancing/non-measurable/measurable); *TBR*_*max*_, maximal [^18^F]FET tumour-to-background ratio; *BV*_*max*_, maximal blood volume (BV); *nBV*_*max*_, normalised maximal BV; *VOL*, lesion volume; *TBR*_*med*_, median [^18^F]FET tumour-to-background ratio; *F*_*med*_, *BV*_*med*_, and *Ki*_*med*_ median blood flow, blood volume and permeability in contrast-enhancing volume

**Fig. 5:**
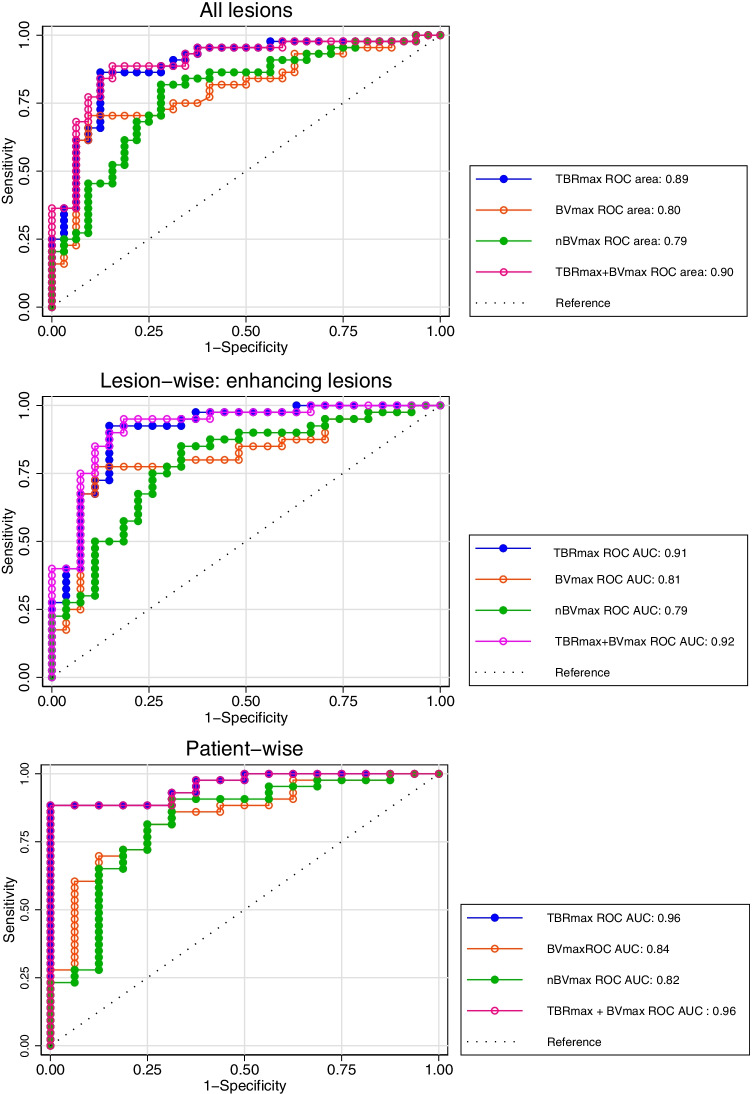
Receiver operating characteristics curve analysis. ROC curves for TBR_max_ (blue), BV_max_ (orange) and nBV_max_ (green) according to lesion- and patient-wise outcome. The pink line shows the ROC curve for the logistic model with both TBR_max_ and either BV_max_ or nBV_max_. ROC AUC for TBR_max_ was significantly (*p* < 0.05) higher than BV_max_ and nBV_max_ except for BV_max_ in enhancing lesions in lesion-wise analysis (*p* = 0.06). ROC AUC for models with both TBR_max_ and either BV_max_ or nBV_max_ were not different from TBR_max_ alone

ROC analyses (Fig. 5) showed higher ROC AUCs for TBR_max_ than both BV_max_ and nBV_max_ in both lesion-wise (all lesions, *p* = 0.04) and in patient-wise analysis (*p* < 0.01), although only borderline significant for BV_max_ in enhancing lesions (*p* = 0.06) and not different in non-enhancing lesions. Combining BV_max_ or nBV_max_ with TBR_max_ did not improve diagnostic performance assessed by ROC AUC. Accordingly, the diagnostic performance at optimal cut-offs (Table 2) was, in general, lower for BV_max_ and nBV_max_ than for TBR_max_. In non-enhancing lesions, optimal cut-off values were lower than in enhancing lesions, and nBV_max_ had the highest specificity, but due to the small number of lesions (*n* = 9), confidence intervals are wide. Subgroup ROC analysis (Supplementary Table S3 in Online resource 1) of enhancing lesions showed similar cut-off and diagnostic performance for TBR_max_ and BV_max_ irrespective of recent RT, IDH and MGMT status, whereas optimal nBV_max_ showed some variability.

**Table 2:** Empirical optimal cut-offs according to ROC analysis

	TBR_max_	BV_max_ (ml/100 g)	nBV_max_	Combined†
Overall (*n* = 76)				
ROC AUC	0.89	0.80	0.79	0.90
Cut-off	2.27	10.43*	6.23	
Sensitivity	86.4(72.6–94.8)	70.5 (54.8–83.2)	81.8 (67.3–91.8)	72.7 (57.2–85.0)
Specificity	87.5 (71.0–96.5)	90.6 (75.0–98.0)	71.9 (53.3–86.3)	90.6 (75.0–98.0)
Odds ratio	44.3 (11.7–166.5)	23.1 (6.2–83.4)	11.5 (3.9–33.6)	25.8 (6.9–94.0)
PPV	90.5 (77.4–97.3)	91.2 (76.3–98.1)	80.0 (65.4–90.4)	91.4 (76.9–98.2)
NPV	82.4 (65.5–93.2)	69.0 (52.9–82.4)	74.2 (55.4–88.1)	70.7 (54.5–83.9)
Enhancing lesions (*n* = 67)				
ROC AUC	0.91	0.81	0.79	0.92
Cut-off	2.27	10.43*	6.75	
Sensitivity	92.5 (79.6–98.4)	77.5 (61.5–89.2)	85 (70.2–94.3)	75 (58.8–87.3)
Specificity	85.2 (66.3–95.8)	85.2 (66.3–95.8)	66.7 (46.6–83.5)	96.3 (81–99.9)
Odds ratio	70.9 (15.1–330)	19.8 (5.6 –69.4)	11.3 (3.6–36.1)	78 (15.5–n.a.)
PPV	90.2 (76.9–97.3)	88.6 (73.3–96.8)	79.1 (64–90)	96.8 (83.3–99.9)
NPV	88.5 (69.8–97.6)	71.9 (53.3–86.3)	75 (53.3–90.2)	72.2 (54.8–85.8)
Non-enhancing lesions (*n* = 9)				
ROC AUC	0.70	0.70	0.90	0.80
Cut-off	1.52	2.28	4.11*	
Sensitivity	75 (19.4–99.4)	75 (19.4–99.4)	75 (19.4–99.4)	75 (19.4–99.4)
Specificity	80 (28.4–99.5)	80 (28.4–99.5)	100 (47.8–100)	100 (47.8–100)
Odds ratio	12 (0.67–na.)	12 (0.67–n.a.)	n.a. (1.81–n.a.)	n.a. (1.81–n.a.)
PPV	75 (19.4–97.4)	75 (19.4–97.4)	100 (29.2–100)	100 (29.2–100)
NPV	80 (28.4–99.5)	80 (28.4–99.5)	83.3 (35.9–99.6)	83.3 (35.9–99.6)
Combined criteria (*n* = 67/9)				
Cut-off (CE / non-CE)	2.27/1.52	10.43*/2.28	6.75/4.11*	
Sensitivity	90.9 (78.3–97.5)	77.3 (62.2–99.4)	84.1(69.9–93.4)	75 (59.7–86.8)
Specificity	84.4 (67.2–94.7)	87.4 (71–96.5)	71.9 (53.3–86.3)	96.9 (83.8–99.9)
Odds ratio	54 (13.7–213)	23.8 (6.9–80.7)	13.5 (4.49–40.6)	93 (14–n.a.)
PPV	88.9 (75.9–96.3)	89.5 (75.2–97.1)	80.4 (66.1–90.6)	97.1 (84.7–99.9)
NPV	87.1 (70.2–96.4)	73.7 (56.9–86.6)	76.7 (57.7–90.1)	73.8 (58–86.1)
Patient-wise (*n* = 59)				
ROC AUC	0.96	0.84	0.82	0.96
Cut-off	2.27	10.43	5.33*	
Sensitivity	88.4 (74.9–96.1)	69.8 (53.9–82.8)	90.7 (77.9–97.4)	86.0 (72.1–94.7)
Specificity	100 (79.4–100)	87.5 (61.7–98.4)	68.8 (41.3–89.)	100 (79.4–100)
Odds ratio	n.a. (26.6–n.a.)	16.2 (3.5–n.a.)	21.5 (5.1–90.5)	n.a. (22.2–n.a.)
PPV	100 (91–100)	93.8 (79.2–99.2)	88.6 (75.4–96.2)	100 (90.5–100)
NPV	76.2 (52.8–91.8)	51.9 (31.9–71.3)	73.3 (44.9–92.2)	72.7 (49.8–89.3)

Estimates are shown with a 95% confidence interval in parenthesis. †Combination of TBR_max_ and best-performing BV cut-offs (indicated with*) *ROC AUC*, area under receiver operating characteristic curve; *PPV*, positive predictive value; *NPV*, negative predictive value; n.a. indicates that values could not be calculated; *CE*, contrast-enhancing lesions

According to the cut-offs determined by ROC analysis, 62 lesions (82%) were concordant positive (*n* = 34) or negative (*n* = 28) (Table 3). A total of 14 lesions were positive on only [^18^F]FET PET (*n* = 11) or only DCE BV (*n* = 3). Among 15 patients with multiple lesions, four lesions were positive on [^18^F]FET PET only and none on DCE BV only. The fraction of progressive lesions increased from 3/28 (11%) in lesions testing negative for both DCE BV and [^18^F]FET PET to 97% in lesions testing positive on both. In lesions testing positive only on [^18^F]FET PET, the fraction with progression was intermediate (7/11 [64%], *p* ≤ 0.01 vs both concordant negative and positive). The fraction of progressive lesion testing positive on only BV also tended to be lower, but the number of lesions (1 out of 3, all with TBR_max_ in the range 1.9–2.1) was too low to assess differences. In the patient-wise analysis, the result of BV imaging did not appear to significantly influence risk as determined by [^18^F]FET PET cut-offs alone. Outcomes in the lesion and patient-wise analysis stratified according to recent radiotherapy are provided in Supplementary Table S4 (see Online resource 1).

**Table 3:** Frequency of progression according to combined optimal empirical cut-off

	BV < cut-off	BV > cut-off	Total
Lesion-wise			
[^18^F]FET < cut-off	3/28 (11%)	1/3 (33%)	4/31 (13%)
[^18^F]FET > cut-off	7/11 (64%)	33/34 (97%)	40/45 (89%)
Total	10/39 (26%)	34/37 (92%)	44/76 (59%)
Patient-wise			
[^18^F]FET < cut-off	3/14 (21%)	2/7 (29%)	5/21 (24%)
[^18^F]FET > cut-off	1/1 (100%)	37/37 (100%)	38/38 (100%)
Total	4/15 (27%)	39/44 (89%)	43/59 (73%)

Numbers refer to fraction (%) with progression

ROC analysis of median parameter values in contrast-enhancing lesion are provided in Table 4 and Supplementary Fig. 1 (see Online resource 1). Overall accuracies (ROC AUC) of F_med_ and BV_med_ were similar, and both were lower than that of TBR_med_, whereas Ki_med_ yielded the lowest accuracy. Combing DCE metrics did not improve ROC AUC compared to BV_max_ alone. No clear influences of recent radiotherapy, MGMT or IDH status were observed in subgroup analyses; see Supplementary Table S3 (Online resource 1).

**Table 4:** ROC analysis median parameters values in CE volumes

	TBR_med_	BV_med_ (ml/100 g)	F_med_ (ml/100 g/min)	Ki_med_ (ml/100 g/min)
ROC AUC	0.91	0.76	0.78	0.68
Cut-off	1.82	2.51	14.2	5.6
Sensitivity	77.5 (61.5–89.2)	65 (48.3–79.4)	92.5 (79.6–98.4)	72.5 (56.1–85.4)
Specificity	92.6 (74.7–99.1)	85.2 (66.3–95.8)	59.3 (38.8–77.6)	66.7 (46–83.5)
Odds ratio	43.1 (9.21–n.a.)	10.68 (3.2 –35.4)	17.9 (4.6–68.1)	5.3 (1.9–15.0)
PPV	93.9 (79.8–99.3)	86.7 (69.3–96.2)	77.1 (62.7–88)	76.3 (59.8–88.6)
NPV	73.5 (55.6–87.1)	62.2 (44.8–77.5)	84.2 (60.4–96.6)	62.1 (42.3–79.3)

Estimates are shown with a 95% confidence interval in parenthesis. *ROC AUC*, area under receiver operating characteristic curve; *PPV*, positive predictive value; *NPV*, negative predictive value

At the 6-month follow-up, five patients had died, all of which were above the patient-wise cut-off for both BV and TBR_max_.

## Discussion

In the present study, we have compared the diagnostic yield of quantitative DCE BV imaging using 2CXM analysis with that of simultaneously performed [^18^F]FET PET imaging in patients with suspected progressive high-grade glioma after standard therapy. The results (Table 2 and Fig. 5) showed good diagnostic performance of maximal BV parameters both lesion-wise and patient-wise with overall accuracies (ROC AUC) of 0.80 and 0.83, respectively, but still lower than [^18^F]FET PET, that showed very good-to-excellent diagnostic performance with ROC AUCs of 0.89 and 0.96, respectively. Combined imaging did not increase diagnostic accuracy compared to [^18^F]FET PET, but the classification-based level of concordance (Table 3) appeared to allow better lesion-wise risk stratification than that of single modalities.

To our knowledge, this is both the first study to investigate the diagnostic performance of 2CXM analysis of DCE data and the first to compare DCE, in general, with amino acid PET, and also one of the largest studies of diagnostic accuracy of DCE imaging in the post-treatment setting. Additionally, attenuation correction in our study was performed with individually acquired low-dose CT making the amino acid PET measurements directly comparable to PET/CT. Previous PET/MRI DSC studies have been performed with either template-based or measured surrogate AC methods that either ignore the effects of surgical metal implants and cranial modifications or are susceptible to artefacts that may impact on measurements [35, 36]. Furthermore, our patient selection criteria are more restrictive than previous studies excluding patients with low-grade glioma, oligodendroglial tumours or non-standard treatment, as the underlying pathology of both tumour and treatment damage, and hence [^18^F]FET PET characteristics, cannot be expected to be identical. This may explain the higher patient-wise accuracy of static parameters in the present study of 0.96 compared to values in the vicinity of 0.70–0.81 in recent studies of unselected glioma populations [21, 25, 37, 38].

MRI perfusion imaging in brain tumours is most widely performed by DSC BV imaging [39, 40]. Meta-analyses of DSC studies have reported pooled sensitivity and specificity of 83–88%, although at variable relative BV cut-offs [6, 41, 42]. Traditionally, DCE imaging has focused on quantification of permeability from a 2-parameter model (Tofts’ model) by estimation of Ve and of K^trans^, a mixed measure of permeability and blood flow. More recent studies have, in general, applied a 3-parameter model (the extended Tofts’ model) also estimating the plasma volume, Vp. The overall sensitivity/specificity/ROC AUC of 77%/84%/0.80 of BV_max_ in the present study is somewhat lower than that of normalised Vp (nVp, 92%/77%/0.87) reported in a mixed population of gliomas and brain metastases following radiotherapy [43]. Of note, diagnostic performance in the glioma subgroup (with only 2/29 with radiation necrosis) was not reported. Other studies have found lower [44, 45] or even no [46] diagnostic value of nVp in suspected recurrent high-grade gliomas. A previous smaller study (*n* = 16) applying Patlak plot analysis of DCE data reported that an enhancing tumour absolute mean BV value of 2.0 ml/100 g provided 100% specificity and sensitivity [47]. Although we could not reproduce this excellent accuracy, it should be noted that the optimal absolute median BV cut-off of 2.5 ml/100 g in CE lesions in the present study is not very different. For clinical use, robustness is also of key importance. Based on the information provided in Fig. 1, the technical failure rate among 413 scans with DCE performed can be estimated to be 1.9% which is acceptable, but slightly higher than for [^18^F]FET PET (0.7%). Our data, thus, suggests that the diagnostic accuracy of BV determined by the 2CXM approach applied is probably within the upper range of prior DCE studies reporting Vp, and also within the range, but not superior to that, of prior DCE and DSC studies in general [6, 41, 42], and may thus provide a viable alternative to other MRI perfusion techniques when amino acid PET is not available.

As opposed to DSC, DCE using 2CXM allows absolute quantification of both blood volume and permeability in addition to blood flow. Although the present analysis has focused on blood volume measurements, which have been more widely applied in clinical glioma imaging, we also assessed the diagnostic yield of other DCE parameters. Blood flow (F) measured by DCE appeared to have a diagnostic accuracy similar to that of BV in enhancing lesions, probably reflecting that the perfusion estimate is dominated by an intravascular flow signal from large- and medium-sized vessels. Quantitative values of Ki had lower diagnostic value than the other DCE metrics for predicting progression. This could reflect that permeability (estimated by Ki or K^trans^) is increased in both progressive tumour and in post-treatment-related effects, and that progressive disease is characterised by an increase in both BV (or Vp) and permeability [43].

Our study confirms very good-to-excellent diagnostic accuracy of [^18^F]FET PET TBR_max_ in recurrent gliomas providing overall accuracies (ROC AUCs) of 0.89 lesion-wise and 0.96 patient-wise. The optimal cut-off of 2.3 in patient-wise analysis and in enhancing lesions was very robust across different subgroups and also within the range of previous studies [17]. Although the number of lesions in the subgroups is too small to make firm conclusions, it is noteworthy that in non-enhancing lesions, the optimal cut-offs of both TBR_max_ and BV parameters are markedly lower than in enhancing lesions, and further that the diagnostic performance of DCE BV was similar to that of [^18^F]FET PET. This could suggest that in lesions with apparently intact BBB, any augmented FET uptake or increased BV, also below standard thresholds, could be indicative of an active tumour. Further studies investigating the potential value of DCE imaging in non-enhancing lesions are warranted.

The present study is among the largest studies of diagnostic yield of simultaneous amino acid PET and MRI BV imaging using hybrid PET/MRI systems. The use of hybrid PET/MRI secures simultaneous measurements and that between-modality variability is not caused by tumour progression, and that measurements are performed under identical physiological conditions and plasma levels of chemotherapy. In agreement with prior smaller studies using DSC, we found that combined BV and [^18^F]FET PET imaging increased specificity compared to [^18^F]FET PET imaging alone [20, 22, 23, 28], although at the expense of lower sensitivity. Similar results have been found for combined [^18^F]FDG PET and DSC BV imaging [43, 48, 49]. In a very recent large retrospective analysis of a heterogeneous sample of 104 patients comprising both low- and high-grade gliomas, the authors also found higher sensitivity of [18]FET PET and higher specificity of DSC BV obtained up to 3 months apart [27]. A large PET/MR study of 105 patients with suspected recurrence of predominantly high-grade gliomas found static [^18^F]FET imaging to provide the highest diagnostic accuracy (79%) along with contrast enhancement (80%) as single modalities, whereas the diagnostic accuracies of both DSC (64%) and spectroscopy (53%) were lower [25]. Other hybrid PET/MRI studies have investigated various combinations of PET and advanced MRI techniques including spectroscopy and/or diffusion-weighted imaging [20, 22, 23, 48, 50, 51]. No single modality is expected to provide 100% accuracy, but as the various imaging modalities depict different aspects of tumour biology, multimodal imaging may provide means to overcome the limitation of single modalities. Although multimodal imaging studies, in general, suggest increased accuracy, the gain in accuracy is often marginal compared to that of the best-performing single modality [52]. Interpretation of multiparametric imaging may be complex, and the optimal combination of modalities and criteria of interpretation has not been established. Here, we have stratified lesions into a 2 × 2 matrix according to simplified single modality cut-offs. Others have applied a simple scoring system [53], or analysis of radiomic features [54] in order to extract and combine information from multiple modalities.

Although perfusion MRI and amino acid PET are often considered complementary modalities, it appears from a decision-making perspective that the incremental value of [^18^F]FET PET added to DCE BV is greater than that of DCE BV added to [^18^F]FET PET, as illustrated by more progressive lesions being positive on only [^18^F]FET PET than only on DCE BV imaging. In many centres, hybrid PET/MR is not available, and perfusion MRI and PET imaging must be obtained on separate MR and PET systems. Although same-day combined evaluation could be achieved by separate scans and subsequent software image registration, the high-positive predictive value of DCE BV shown here could suggest a sequential imaging strategy with [^18^F]FET PET only required if DCE BV is negative. Based on data presented in Table 3, 37/76 (49%) of lesions and 44/59 (75%) of patients would accordingly not require [^18^F]FET PET and thus reduce overall costs substantially at the expense of a minor decrease in the fraction of correctly classified lesions to 86.7% (sensitivity 93.2% and specificity 78.1%) compared to [^18^F]FET PET (88.2%) in all lesions. This is in line with a recent study of DSC BV and FET PET, suggesting that such a strategy could reduce the need for [.^18^F]FET PET by 42%. [27]

Similar to others, we applied a combined outcome measure based on a follow-up including histology and MRI follow-up. The present study lacks histopathological confirmation in 2/3 of lesions, which may be seen as a limitation. However, retrospective data that select only patients with tissue as a reference may suffer from verification bias, as the decision to resect or biopsy may be influenced by the PET result, among other factors [18]. In treated tumours, both imaging and tissue samples may represent heterogeneous pathology with both tumour and treatment effects. In the absence of image-correlated stereo-biopsies, it is not possible directly to link the tissue examined with imaging features within the lesion. Also, the study population may differ from that of routine imaging and also from studies involving MR only which may allow consecutive imaging without referral bias, as opposed to PET/MR relying on the fraction of patients not managed by MRI alone. To ensure a homogenous study population, we applied relatively strict criteria in terms of histology and prior treatment and furthermore only included patients with evaluable outcomes after the scan, thus excluding also those in whom second-line treatment was initiated after the scan without histopathological confirmation. The imaging protocol did not include dynamic [^18^F]FET PET imaging which has been reported to improve diagnostic accuracy [20, 38]. However, the added value of dynamic imaging may be modest [20, 55], and as dynamic characteristics may be related to tumour perfusion [56, 57], we expect that the incremental diagnostic value of dynamic imaging would be relatively small when added to combined static [^18^F]FET PET and perfusion imaging. Finally, the present analysis was based on simplified metrics not taking into account morphology or prior imaging, which are key elements of clinical reading. Due to these limitations, diagnostic accuracies reported here should be interpreted with caution, and future prospective studies should investigate the diagnostic performance in a well-controlled, multi-reader set-up. Still, we believe that the comparison of diagnostic performance between modalities is valid due to the common outcome measure.

## Conclusions

The present study shows a good diagnostic performance of DCE BV imaging using the 2CXM approach with maximal BV providing the highest accuracy among investigated metrics, but also confirms higher overall diagnostic accuracy of [^18^F]FET PET for differentiation of tumour progression from treatment-related effects in patients with anaplastic astrocytoma and glioblastoma. Combined imaging did not improve the diagnostic accuracy of [^18^F]FET PET, but may increase specificity and allow better risk stratification of [^18^F]FET PET avid lesions.

## Supplementary Information

Below is the link to the electronic supplementary material.

Supplementary file1 (DOCX 172 KB)

Supplementary file2 (XLSX 17 KB)

## Data Availability

Meta-data are available as Supplementary information in Online resource 2. Additional used and/or analysed during the current study are available from the corresponding author on reasonable request.
