# Supplementary material for: Diagnostic yield of simultaneous dynamic contrast-enhanced magnetic resonance perfusion measurements and [18F]FET PET in patients with suspected recurrent anaplastic astrocytoma and glioblastoma
Source: Eur J Nucl Med Mol Imaging. 2022 Jul 30;49(13):4677–91. doi: 10.1007/s00259-022-05917-3 (PMC9605929; doi:10.1007/s00259-022-05917-3)
Supplement: Supplementary file 1 — Supplementary file1 (DOCX 172 KB) [file 259_2022_5917_MOESM1_ESM.docx]

**Online Resource 1.**

**Supplementary methods**

*Details of imaging protocol*

The PET images were reconstructed into a 344x344 matrix (voxel-size 0.8.x0.8x2 mm^3^) using 3D OP-OSEM (4 iterations, 21 subset) and applying a 5 mm Gaussian filter. The spatial resolution of the system is approx. 5 mm (1). Attenuation correction was performed either using a separately obtained low-dose CT (120 kV, 30 mAs, 5 mm slice width, Siemens Biograph PET/CT system) as previously described (2) or MRI based attenuation correction from a region specific optimization of an UTE sequence (RESOLUTE) (3). Structural MRI included as a minimum axial T2 BLADE (0.7x0.7x5 mm^3^), T2 FLAIR (1.2x0.9x5 mm^3^) and post-contrast 3D-T1 (1x1x1 mm^3^) weighted sequences.

Dynamic T1 weighted imaging was performed using a fast 3D spoiled gradient echo (VIBE) sequence with full brain coverage (2.4x2.4x5 mm^3^, TR/TE 2.94/0.86 sec, flip angle 14 degrees). Images for T1-mapping were acquired before contrast injection using variable flip angles (4, 8, 14 and 20 or from Jan 2019: 2, 3, 4, 6 and 8 degrees) and otherwise identical parameters. A total of 180 frames with a temporal resolution of 2.6 sec were acquired during a double bolus passage of 0.05 mmol/kg (Gadovist ® 1 mmol/ml, Bayer, Berlin, Germany) injected at 18 and 85 s after the dynamic DCE acquisition was started using a power injector (Medrad, Pittsburgh, PA) at a rate of 3 ml/sec followed by 10 ml of NaCl (3ml/sec).

1. Delso G, Furst S, Jakoby B, Ladebeck R, Ganter C, Nekolla SG, et al. Performance measurements of the Siemens mMR integrated whole-body PET/MR scanner. J Nucl Med. 2011;52(12):1914-22.

2. Andersen FL, Ladefoged CN, Beyer T, Keller SH, Hansen AE, Hojgaard L, et al. Combined PET/MR imaging in neurology: MR-based attenuation correction implies a strong spatial bias when ignoring bone. Neuroimage. 2014;84:206-16.

3. Ladefoged CN, Andersen FL, Kjaer A, Hojgaard L, Law I. RESOLUTE PET/MRI Attenuation Correction for O-(2-(18)F-fluoroethyl)-L-tyrosine (FET) in Brain Tumor Patients with Metal Implants. Front Neurosci. 2017;11:453.

**Supplementary tables and figures**

**Suppl. Table S1. Standard post-operative therapy for high grade gliomas**

| **Tumor** | **Radiotherapy** | Adjuvant TMZ |
| --- | --- | --- |
| Anaplastic astrocytoma, IDH mutated: | 59.4 Gy/33 fractions | 12 series |
| GBM  Anaplastic astrocytoma, IDH wildtype | 60 Gy/30 fractions with concomitant TMZ | 6 series |

Based on current Danish guidelines (available from www.dnog.dk)

**Suppl. Table S2. Lesion summary statistics according to verification**

|  | Histological verification  n=28 | Clinical/radiological  follow-up  n=33 |
| --- | --- | --- |
| Day, last surgery | 248 (27-2167) | 195 (15-1041) |
| Days from RT | 231 (56-2877) | 255 (67-4494) |
| GBM, n (%) | 26 (93) | 41 (85) |
| IDH wildtype, n (%) | 24 (86) | 40 (83) |
| MGMT methylated, n (%) | 9 (32) | 33 (69)‡ |
| RANO group | 1/6/21 | 8/26/14‡ |
| TBR_max_ | 3.0 (1.9-5.0) | 1.8 (0.3-4.2)‡ |
| BV_max_ (mL/100g) | 13.9 (1.2-84.0) | 4.3 (0.2-106.3)‡ |
| nBV_max_ | 21.2 (1.6-204.7) | 5.6 (0.5-166.6)‡ |
| VOL_FET_ (ml) | 12.3 (0.9-46.4) | 0.4 (0-38.5)‡ |
| VOL_BV_ (ml) | 2.6 (0-33.8) | 0.1 (0-11.4)‡ |
| VOL_CE_ (ml) | 3.1 (0-32) | 0.35 (0-16.9)‡ |
| TBR_med_* | 2.1 (1.4-3.1) | 1.5 (0.3-2.8)‡ |
| F_med_ (mL/100g/min) * | 23.4 (11.2-73.4) | 15.9 (3.1-72.1)‡ |
| BV_med_ (mL/100g) * | 3.2 (0.4-14.7) | 2.0 (0.1-11.9)‡ |
| Ki_med_ (mL/100g/min) * | 7.1 (0.2-40) | 4.5 (0-40)‡ |

‡ p<0.01, * from contrast enhancing volume only; RT radiotherapy; GBM glioblastoma; IDH isocitrate dehydrogenase; MGMT methylguanine-DNA-methyltransferase; RANO group (non-enhancing/non-measurable/measurable);TBR_max_ maximal FET tumor-to-background ratio; BV_max_ maximal blood volume (BV), nBV_max_ normalized maximal BV; VOL lesion volume; TBR_med_ median tumor-to-background ratio and F_med_, BV_med_ and Ki_med_ median blood flow, blood volume and permeability within enhancing volume.

**Suppl. Table S3. Subgroup ROC analyses in contrast enhancing lesions**

|  | All CE lesions | Recent RT | No recent RT | MGMT  non-meth | MGMT meth | IDH wt |
| --- | --- | --- | --- | --- | --- | --- |
|  | n=67 | n=29 | n=47 | n=34 | n=42 | n=64 |
| **TBR_max_** |  |  |  |  |  |  |
| ROC AUC | 0.906 (<0.001) | 0.989 (<0.001) | 0.880 (<0.001) | 0.911 (<0.001) | 0.878 (<0.001) | 0.883 (<0.001) |
| Cut-off | 2.266 | 2.229 | 2.275 | 2.215 | 2.300 | 2.266 |
| Sens/Spec | 0.925 / 0.852 | 0.941 / 1.000 | 0.913 / 0.750 | 0.958 / 0.875 | 0.875 / 0.842 | 0.914 / 0.818 |
| **BV_max_** |  |  |  |  |  |  |
| ROC AUC | 0.811 (<0.001) | 0.834 (<0.001) | 0.807 (<0.001) | 0.865 (<0.001) | 0.796 (<0.001) | 0.826 (<0.001) |
| Cut-off | 10.435 | 10.790 | 10.265 | 10.265 | 13.775 | 10.435 c |
| Sens/Spec | 0.775 / 0.889 | 0.706 / 1.000 | 0.826 / 0.813 | 0.792 / 1.000 | 0.750 / 0.895 | 0.800 / 0.909 |
| **nBV_max_** |  |  |  |  |  |  |
| ROC AUC | 0.786 (<0.001) | 0.856 (<0.001) | 0.785 (<0.001) | 0.760 (0.019) | 0.776 (0.001) | 0.777 (<0.001) |
| Cut-off | 6.746 | 5.431 | 9.709 | 5.530 | 13.688 | 12.062 |
| Sens/Spec | 0.850 / 0.667 | 0.824 / 0.818 | 0.870 / 0.625 | 0.917 / 0.625 | 0.750 / 0.789 | 0.771 / 0.727 |
| **TBR_med_** |  |  |  |  |  |  |
| ROC AUC | 0.910 (<0.001) | 0.973 (<0.001) | 0.875 (<0.001) | 0.901 (<0.001) | 0.911 (<0.001) | 0.897 (<0.001) |
| Cut-off | 1.824 | 1.548 | 1.974 | 1.669 | 1.824 | 1.824 |
| Sens/Spec | 0.775 / 0.926 | 0.941 / 0.909 | 0.696 / 0.938 | 0.833 / 0.875 | 0.750 / 0.947 | 0.800 / 0.909 |
| **F_med_** |  |  |  |  |  |  |
| ROC AUC | 0.760 (<0.001) | 0.805 (0.002) | 0.739 (0.010) | 0.695 (0.169) | 0.799 (<0.001) | 0.750 (0.001) |
| Cut-off | 14.200 | 16.665 | 14.200 | 18.355 | 14.210 | 15.920 |
| Sens/Spec | 0.925 / 0.593 | 0.765 / 0.818 | 0.957 / 0.563 | 0.750 / 0.750 | 0.938 / 0.684 | 0.886 / 0.636 |
| **BV_med_** |  |  |  |  |  |  |
| ROC AUC | 0.781 (<0.001) | 0.727 (0.027) | 0.826 (<0.001) | 0.742 (0.015) | 0.809 (<0.001) | 0.753 (<0.001) |
| Cut-off | 2.515 | 1.435 | 2.515 | 2.520 | 1.435 | 2.515 |
| Sens/Spec | 0.650 / 0.852 | 0.882 / 0.545 | 0.783 / 0.813 | 0.708 / 0.750 | 0.938 / 0.632 | 0.657 / 0.818 |
| **Ki_med_** |  |  |  |  |  |  |
| ROC AUC | 0.675 (0.014) | 0.663 (0.187) | 0.694 (0.029) | 0.646 (0.262) | 0.679 (0.062) | 0.686 (0.015) |
| Cut-off | 5.555 | 5.555 | 5.440 | 6.560 | 5.640 | 5.555 |
| Sens/Spec | 0.725 / 0.667 | 0.706 / 0.727 | 0.739 / 0.625 | 0.625 / 0.750 | 0.750 / 0.737 | 0.743 / 0.636 |

ROC AUC area under receiver operating characteristics curve; RT radiotherapy; IDH isocitrate dehydrogenase; MGMT methylguanine-DNA-methyltransferase; RANO group (non-enhancing/non-measurable/measurable); TBR_max_ maximal FET tumor-to-background ratio; BV_max_ maximal blood volume (BV), nBV_max_ normalized maximal BV; VOL lesion volume; TBR_med_ median tumor-to-background ratio and F_med_, BV_med_ and Ki_med_ median blood flow, blood volume and permeability within enhancing volume.

**Suppl. Table S4. Outcome according to recent radiotherapy (< 6 month)**

| **Lesion-wise** | **Recent RT** | **(n=29)** |  |  | **No recent RT** | **(n=47)** |  |
| --- | --- | --- | --- | --- | --- | --- | --- |
|  | **BV < cutoff** | **BV > cutoff** | **Total** |  | **BV < cutoff** | **BV > cutoff** | **Total** |
| **[^18^F]FET < cutoff** | 1/12 (8%) | 0/0 (-) | 1/12(5%) |  | 2/16 (13%) | 1/3 (33%) | 3/19(16%) |
| **[^18^F]FET > cutoff** | 4/4 (100%) | 13/13 (100%) | 17/17 (100%) |  | 3/7 (43%) | 20/21 (95%) | 23/28 (82%) |
| **Total** | 5/16 (31%) | 13/13 (100%) | 18/29 (62%) |  | 5/23 (22%) | 21/24 (88%) | 26/47 (55%) |
|  |  |  |  |  |  |  |  |
| **Patient-wise** | **Recent RT** | **(n=24)** |  |  | **No recent RT** | **(n=37)** |  |
|  | **BV < cutoff** | **BV > cutoff** | **Total** |  | **BV < cutoff** | **BV > cutoff** | **Total** |
| **[^18^F]FET < cutoff** | 1/6 (17%) | 1/3 (33%) | 2/9 (22%) |  | 2/8 (25%) | 1/4 (25%) | 3/12(25%) |
| **[^18^F]FET > cutoff** | 1/1 (100%) | 12/12 (100%) | 13/13 (100%) |  | 0/0 (-) | 25/25 (100%) | 25/25 (100%) |
| **Total** | 2/8 (25%) | 13/15 (87%) | 15/22 (68%) |  | 2/8 (25%) | 26/39 (90%) | 28/37 (76%) |

Numbers refer to fraction (%) with progression, BV blood volume, RT radiotherapy.


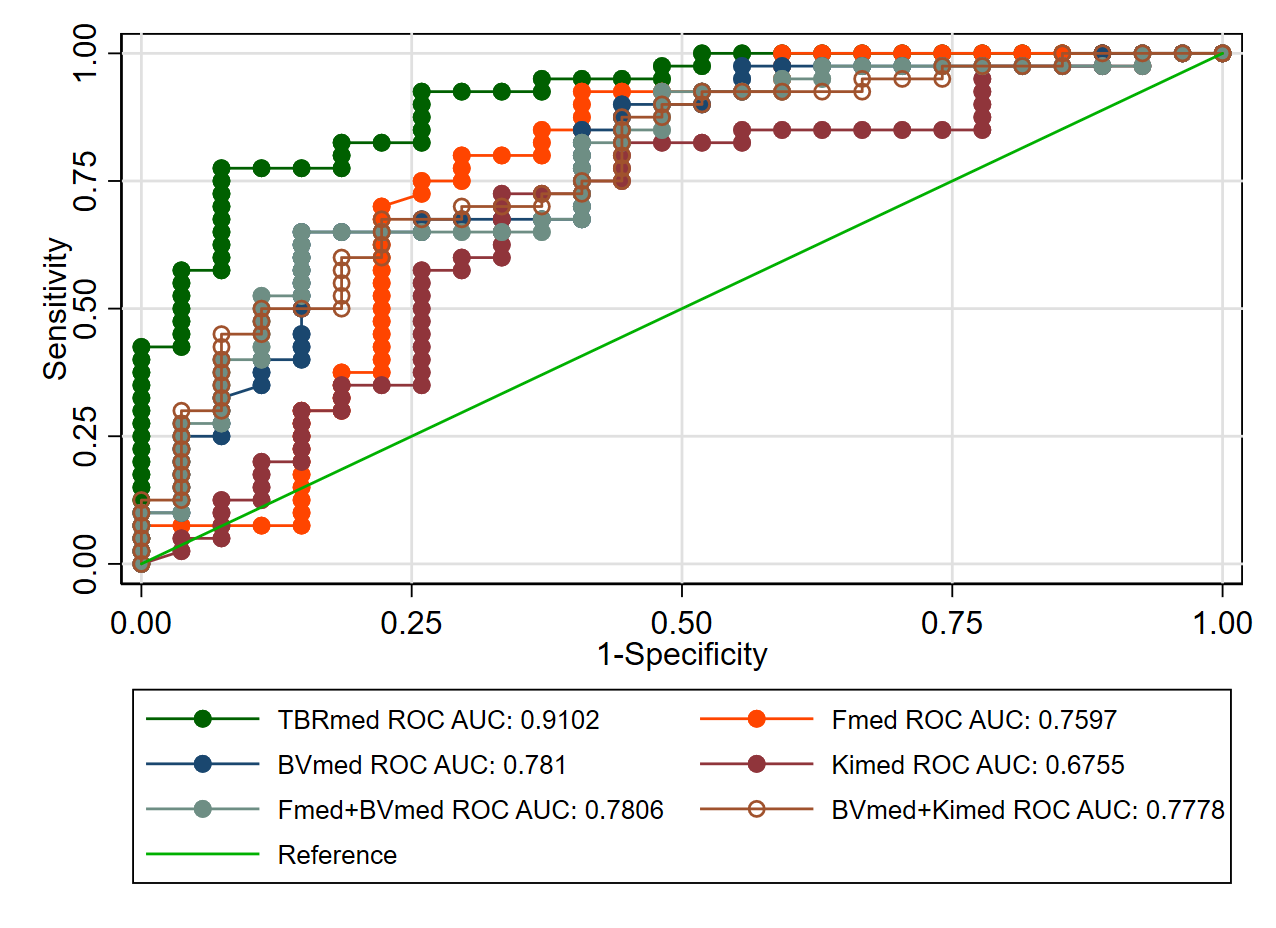


|  | TBR_max_ | F_med_ | BV_med_ | Ki_med_ |
| --- | --- | --- | --- | --- |
| TBR (1) |  | 0.0476 | 0.0419 | 0.0004 |
| F_med_ |  |  | 0.6446 | 0.4182 |
| BV_med_ |  |  |  | 0.2480 |
| BV_med_+F_med_ | 0.0377 |  | 0.9552 |  |
| BV_med_+Ki_med_ | 0.0290 |  | 0.8499 |  |

**Suppl. Fig S1. ROC AUC of median TBR and DCE metrics in enhancing volumes.** P-values for comparison of ROC AUCs shown in inserted table.
